# Toward the Characterization of Human Pro-Resolving Macrophages?

**DOI:** 10.3389/fimmu.2020.593300

**Published:** 2020-11-13

**Authors:** Philippe Saas, Cécile Chagué, Mélissa Maraux, Thomas Cherrier

**Affiliations:** Univ. Bourgogne Franche-Comté, INSERM, EFS BFC, UMR1098, Interactions Hôte-Greffon-Tumeur/Ingénierie Cellulaire et Génique, Fédération Hospitalo-Universitaire INCREASE, LabEx LipSTIC, Besançon, France

**Keywords:** macrophage, Liver X receptor, Arginase-1, inflammation, efferocytosis, apoptotic cell, human, mouse

## Introduction

Macrophages are a heterogeneous cell population with a high plasticity. They may arise from hematopoietic progenitors during embryogenesis and become tissue-resident macrophages (TRM) with a high capacity to self-renew. Alternatively, they may differentiate from blood monocytes during inflammation (1). These innate immune cells may mediate either pro- or anti-inflammatory functions (1) and play a critical role during the resolution phase of inflammation. In this setting, they shift from a pro-inflammatory toward a pro-resolving profile (1). This transition, named macrophage reprogramming, is triggered by apoptotic cell elimination [a process called efferocytosis (2–4)], as well as by extracellular vesicles released by apoptotic cells (5). Together with signals provided by cognate receptors recognizing apoptotic corpses, apoptotic cell-derived materials (i.e., nucleic acid, lipids, amino acids, and intermediate metabolites) participate in this reprogramming by modifying macrophage metabolism (6–11). At steady state, efferocytosis imprints an anti-inflammatory profile in mouse TRM (12, 13). The tissue microenvironment affects this anti-inflammatory program of mouse macrophages residing in cavities by inducing the apoptotic cell recognition receptor, TIM-4, and down-regulating Toll-like receptor (TLR) 9 (14). Based on 3 recently published manuscripts (10, 15, 16) that bring significant contributions to human macrophage characterization and shed light on discrepancies between mouse and human resolving macrophages, we here comment and discuss this interspecies variability. Today, while mouse pro-resolving macrophages and the efferocytosis process begin to be deciphered, data on human macrophages remain scarce. Unraveling human pro-resolving macrophages may lead to their identification in diseases and the development of innovative therapeutic approaches (17). This review will evoke a main difference already identified between mouse and human macrophages, the L-arginine metabolism. Then, we will consider an anti-inflammatory pathway in mouse macrophages (7), the Liver X receptor (LXR) pathway that may be rather pro-inflammatory in human macrophages. Finally, we will propose possible explanations for these differences and potential solutions to identify signaling molecules and/or metabolites governing or characterizing human pro-resolving macrophages.

## Arginine Metabolism, a Well-Described Pathway Differing Between Human and Mouse Macrophages

Macrophages represent heterogeneous cells, even in a given tissue, at steady state or during pathogenic situations. This heterogeneity lies on the macrophage origins, but also on their localization (1). Macrophages are highly plastic cells; they may exert a vast “spectrum” of functions characterized by an array of different macrophage phenotypes/subtypes (18). The two extreme polarized phenotypes of this continuum are called M1 and M2. M1 represent pro-inflammatory (“classically” activated) macrophages involved in anti-infectious responses. M2 are anti-inflammatory (“alternatively” activated) macrophages, which can be subdivided into several subtypes with different functions: immunosuppressive tumor-associated macrophages (TAM), pro-resolving macrophages, but also macrophages associated with T helper 2 (Th2) responses found in asthma for instance. Type 2 cytokines (e.g., IL-4) are involved in M2 macrophage polarization. Response to IL-4 may differ between mouse and human macrophages (19, 20). This M1/M2 dichotomy is known to be associated with a distinct arginine metabolism, in particular for mouse macrophages (21, 22). M1 macrophages metabolize arginine to generate nitric oxide (NO) contributing to pathogen killing. In contrast, mouse M2 macrophages convert the same substrate to produce ornithine, and then polyamines (e.g., putrescine). Polyamines participate in collagen synthesis necessary for tissue repair. This implies two different enzymes, inducible NO synthase (iNOS, also known as NOS2) and arginase-1, respectively (23).

**Figure 1:**
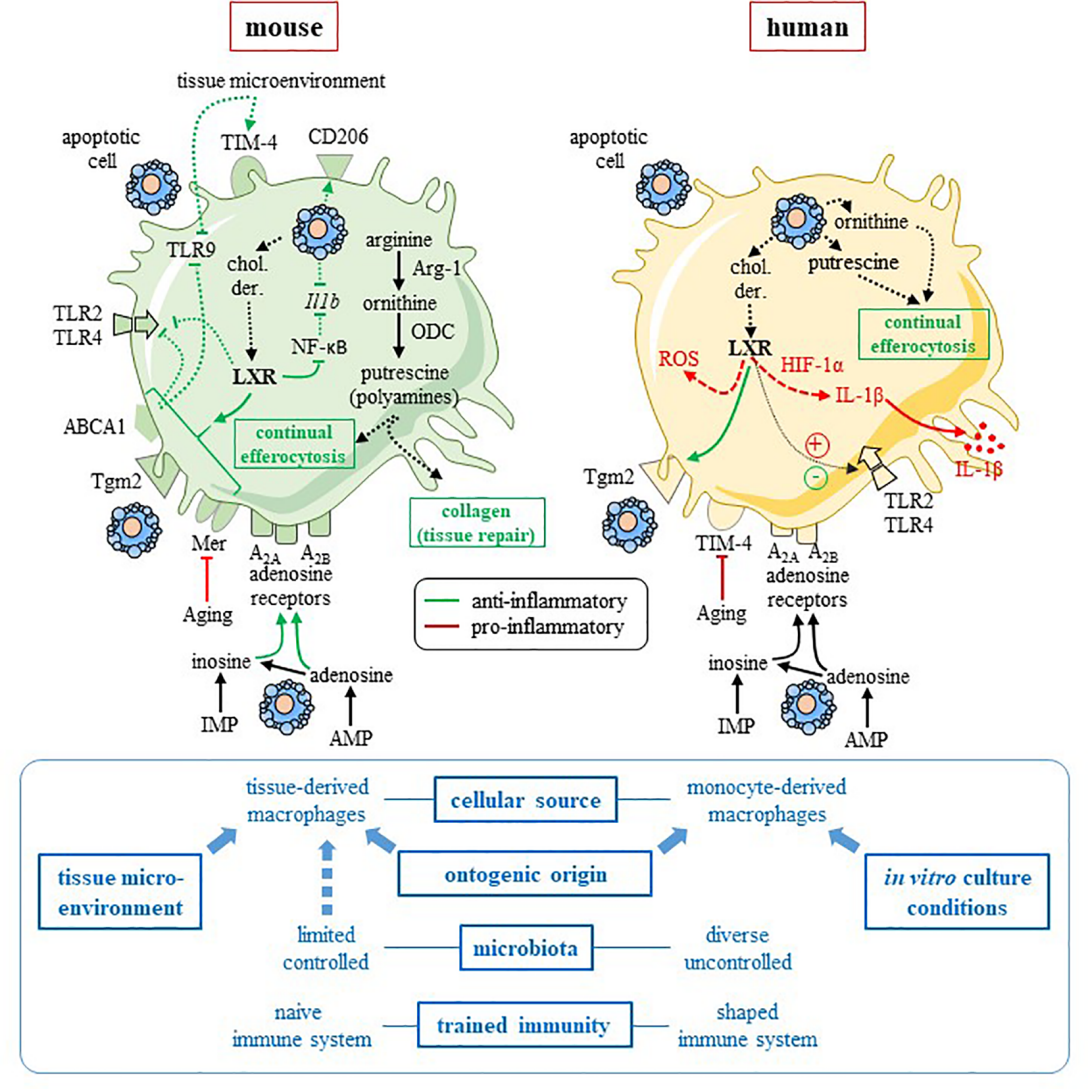
Comparison of mouse and human pro-resolving macrophages. This figure summarizes the data discussed in the text, and focuses on two pathways involved in mouse macrophage anti-inflammatory reprogramming, namely the L-arginine metabolism and the LXR pathway. Interspecies heterogeneity is reported for these two pathways. Mouse pro-resolving macrophages (left-hand side) are characterized by arginase-1 (Arg-1) and uses the arginine pathway to stimulate tissue repair and “continual” efferocytosis. Efferocytosis and tissue microenvironment imprint an anti-inflammatory profile with an increased expression of M2 receptor CD206 (13) and efferocytic receptor TIM-4 (14), as well as a downregulation of pro-inflammatory *Tlr9* (14) and *Il1b* (13) genes. Cholesterol derivatives (chol. der.) issued from apoptotic cells may promote the LXR pathway that is responsible for upregulation of efferocytic receptor Mer (7) and an anti-inflammatory response, including inhibition of NF-κB (31) and TLR2, 4 and 9 signaling pathway *via* the LXR target factor, ABCA1 (32). LXR upregulates Tgm2 in mouse macrophages (33). Metabolites released from apoptotic cells (i.e., inosine-monophosphate and adenosine-monophosphate) imprint an anti-inflammatory profile (11), possibly *via* A_2A_ and A_2B_ adenosine receptors, which are highly expressed and functional in mouse cells. Thus, in mouse macrophages, adenosine A_2A_ receptors are the primary target of apoptotic cell-derived adenosine and these receptors mediated apoptotic-cell induced immune suppression (34, 35). Aging affects efferocytosis efficacy by downregulating Mer receptor (36). The picture for human macrophages (right-hand side) is more complicated and data remain incomplete. While apoptotic-cell derived ornithine and putrescine participates in “continual” efferocytosis and the anti-inflammatory program of human pro-resolving macrophages (10). LXR activation leads to anti-inflammatory functions [with upregulation of efferocytic receptor Tgm2 (37) and inhibition of TLR4 signaling (38)] and pro-inflammatory functions (15, 16, 38) [with the production of IL-1β *via* HIF-1α (15), as well as ROS (38)]. Human cells are less receptive to inosine and adenosine with reduced expression and function of adenosine receptors in comparison to mouse cells (39). Aging disturbs efferocytosis efficacy and the resolution of inflammation by downregulating TIM-4 receptor (36). Factors that may explain interspecies differences are written in blue font on the bottom of the figure. Blue arrows mean an influence of a given factor. Some of these factors affect rather mouse macrophages (i.e., tissue microenvironment) and others human macrophages (e.g., *in vitro* culture conditions). **→** (or plus) and ⊣ (or minus) symbols mean stimulation and inhibition, respectively. Red color means pro-inflammatory and green color pro-resolving. Solid line identifies a direct effect, while dotted line an indirect (or supposed) effect. ABCA1, adenosine triphosphate-binding cassette A1; AMP, adenosine-monophosphate; Arg-1, arginase-1; chol. der., cholesterol derivatives; HIF-1α, hypoxia-inducing factor-1α; *Il1b*, *interleukin-1* beta gene; IL-1β, interleukin-1β; IMP, inosine-monophosphate; LXR, Liver X receptor; NF-κB, nuclear factor-kappa B; ODC, ornithine decarboxylase; ROS, reactive oxygen species; Tgm2, transglutaminase-2; TIM4, T-cell immunoglobulin and mucin domain containing 4; TLR, Toll-like receptor. This figure was depicted, in part, by using Servier Medical Art, https://smart.servier.com/.

Interspecies differences have been reported for these two enzymes with a functional expression in mouse macrophages, but not in human macrophages (10, 23–30). Arginase-1 is considered as a marker for anti-inflammatory mouse macrophages (**Figure 1**), but may be absent in human pro-resolving macrophages (10, 24, 27). Several hypotheses may explain this discrepancy (see (22) and *Discussion*). The main hypothesis is the cellular source of macrophages that is most frequently *in vitro* monocyte-derived macrophages (MDM) in humans, whereas already differentiated macrophages are isolated from mice (22). However, mouse macrophages isolated from different tissues exhibit more difference than similarities in their transcriptomic program (40). Nevertheless, this difference in macrophage arginine metabolism is also found in other mammalian species. Rat macrophages behave as mouse (41), while Syrian hamster (41, 42), monkey (25), pig macrophages (29), even badger and ferret macrophages (43) exhibit the same arginine metabolism as human MDM. This is observed even with the same cellular source (i.e., alveolar macrophages) (25, 41). Non-mammalian M1/M2 macrophages, such as those of the European common carp (44), metabolize arginine strictly as mouse macrophages.

Considering human pro-resolving macrophage, arginase-1 and arginine metabolism are not required for “continual” efferocytosis [i.e., the capacity to maintain efferocytosis after the first ingestion (10)]. Indeed, apoptotic cell-derived ornithine and the putrescine pathway contribute to this “continual” efferocytosis, a critical step for macrophage reprogramming (10) (**Figure 1**).

Overall, based on these data (i.e., L-arginine metabolism), interspecies heterogeneity exists between human and mouse pro-resolving macrophages. Whereas different reasons could explain these results (see Discussion), this renders difficult to transpose simply data obtained in mouse macrophages to humans and justifies the need to study human pro-resolving macrophages. Finally, some arginase isoforms may be also pro-inflammatory (45), and this may explain the absence of this enzyme in human pro-resolving macrophages.

## Liver X Receptor Signaling, Another Pathway Differing Between Human and Mouse Macrophages

The other difference existing between human and mouse macrophages is related to macrophage reprogramming, and efferocytosis regulation. Digestion of lipids derived from apoptotic cells by macrophages and lipid metabolism are critical for proper efferocytosis (including “continual” efferocytosis) and mouse macrophage reprogramming (7–9). Digestion of apoptotic cell-derived lipids leads to an increase of cholesterol derivatives and fatty acids which trigger LXR and peroxisome proliferator-activated receptor (PPAR), respectively (46). These nuclear receptors induce the increased expression of efferocytic receptors (e.g., Mer), and the release of soluble bridging molecules (e.g., MFG-E8) facilitating the binding of apoptotic cells (46). Thus, LXR activation exerts functions that may explain some of the anti-inflammatory functions acquired by mouse macrophages after efferocytosis (**Figure 1**).

Macrophages express the LXRα isoform restricted to cells with high cholesterol turnover and the ubiquitous isoform, LXRβ (31). These receptors act as cholesterol sensors to regulate intracellular cholesterol and lipogenesis (31). Prior LXR stimulation in murine macrophages prevents TLR4 activation induced by lipopolysaccharide (LPS) (47, 48). It inhibits LPS-induced expression of inflammatory genes, such *Nos2* or *Il6* genes (47). LXR activation interferes with TLR signaling (i.e., TLR2, 4 and 9) *via* the adenosine triphosphate-binding cassette A1 (ABCA1) transporter that dampens the recruitment of the adaptor MYD88 (32). Moreover, LXR activation inhibits inflammatory responses by antagonizing the pro-inflammatory transcription factor, NF-κB (31). However, this corresponds mainly to data obtained in mice (**Figure 1**). This is sufficient to state that the LXR pathway is anti-inflammatory. Data on LXR anti-inflammatory functions exist in human macrophages with the increase of transglutaminase-2 (Tgm-2) expression after efferocytosis (37). Tgm-2 is also upregulated by LXR in mouse macrophage (33) and stimulates efferocytosis by stabilizing the interaction between the phagocytic receptor β3 integrin, the bridging molecule MFG-E8 and the apoptotic corpse (49) (**Figure 1**). Thus, LXR activation by apoptotic cell-derived materials may participate in anti-inflammatory “continual” efferocytosis.

On the contrary, long-term LXR activation (i.e., 48 h) potentiates the LPS pro-inflammatory response in human MDM, while short-term LXR activation reduces the LPS responses (38). This suggests a different response to LXR activation after efferocytosis in human macrophages. Two recent manuscripts convincingly challenge the anti-inflammatory role of LXR by demonstrating that LXR stimulation is rather a pro-inflammatory signal in human MDM with increased IL-1β production (**Figure 1**) (15, 16). Human and mouse macrophages -derived in culture using M-CSF- are compared in different experiments, and these cells do not respond similarly to LXR activation (15). Overall, the engagement of LXR signaling pathway in mouse macrophages stimulates macrophage reprogramming after efferocytosis leading to a pro-resolving profile. In contrast, LXR activation may lead to a more complex and diverse response in human macrophages, not always associated with a resolution of inflammation.

## Discussion

These two examples illustrate the differences existing between mouse and human macrophages. Comparison of myeloid cell infiltrates in human lung cancers and in corresponding mouse models using single cell transcriptomic analysis highlights this species difference (50). Mouse and human TAM exhibit a different signature, whereas a comparable signature is found for neutrophils, monocyte, and dendritic cell subsets of both species (50). Discrepancies exist also in the efferocytosis machinery of macrophages with aging. Aging induces a reduced Mer expression in mouse macrophages, while a low TIM-4 expression is observed in human macrophages from elderly individuals (36). Apoptotic-cell derived metabolites control mouse macrophage reprograming toward a pro-resolving profile (11). These metabolites are released similarly by both human and mouse apoptotic cells (11). Among them, two metabolites signal *via* A_2A_ and A_2B_ adenosine receptors. Species differences have been reported for adenosine receptors, with human cells being less responsive than mouse cells (39). This predicts that human macrophages could be less sensitive to the anti-inflammatory effects of these metabolites. This supports the specificity of human pro-resolving macrophages. But how?

To answer this question, we first try to explain the species differences based on macrophage biology. Then, we propose some solutions to limit these differences. This may be useful to study human pro-resolving macrophages in more (patho) physiological conditions, and to compare these cells to their mouse counterpart and use powerful experimental models.

The main explanation of the species difference is the cellular source of macrophages with the principal source of human cells being blood monocytes *versus* tissue-derived mouse macrophages (22). The ontogenic origin (i.e., differentiated monocytes *versus* hematopoietic progenitors) may affect macrophage phenotype and function (1). The local microenvironment plays also a role in imprinting macrophage phenotype and function (14). However, some studies used the same cellular source with still interspecies differences (25, 41).

Another explanation is the *in vitro* culture step/system necessary to obtain human macrophages (22). Different cytokine cocktails influence significantly macrophage phenotypes (18, 51). Again, the use of the same protocols for both mouse and human cells does not always lead to the same function (15). A study has reported that human macrophages generated from monocytes using M-CSF (i.e., the classical model used to obtain human macrophages) do not reflect *in vivo* pro-resolving macrophages. On the contrary, monocytes kept for 24 h in culture after isolation better recapitulate these phagocytes present in spontaneous pro-resolving skin lesions (36). This represents an interesting track. Culture conditions are used to mimic the *in vivo* situations. This may be achieved through 3-dimensional cultures reflecting the *in vivo* microenvironment with extracellular matrix interactions (52). *In vitro* models of resolution have been also set-up with sequential exposure to a complex mixture of factors (i.e., chemokine, bacterial vesicles, and cytokines) (53). Whether it influences macrophage anti-inflammatory function remains to be determined. Macrophages require their local microenvironment and signals to maintain their *in vivo* phenotype (22).

Finally, mice are housed under specific pathogen-free (SPF) conditions since their birth, and exhibit a naive immune system more similar to neonatal humans (54). In contrast, humans live in a pathogen rich-environment that shapes their immune system. Two major parameters may affect differently mouse and human pro-resolving macrophages, namely, microbiota and trained immunity. Microbiota and their metabolites influence pro-resolving macrophages. Laboratory mice born to wild mice may correct this bias (55). Controlled infections of laboratory SPF mice with “bystander” pathogens (i.e., herpesviruses) may also limit this bias by humanizing immunological responses in mice (54). Trained immunity describes an immunological memory of innate immune cells (including macrophages), associated with a long-term functional reprogramming due to epigenetic modifications, including histone modification or DNA methylation (56). This reprogramming, induced by inflammatory signals, leads to an altered response towards a second challenge (56). This may either enhance (e.g., BCG vaccination) or inhibit (e.g., LPS and the immune paralysis observed in sepsis) macrophage functions (56). Human macrophages may correspond to trained cells, while mouse cells are naive. LXR activation triggers epigenetic modifications at human pro-inflammatory genes (e.g., *IL6*) stimulating their expression (16). LXR activation may induced trained immunity. An epigenetic modification may explain the interspecies differences of the arginine metabolism (30).

In conclusion, interspecies variability affects certain pathways involved in macrophage reprogramming (**Figure 1**). It is necessary to study specifically human pro-resolving macrophages in *in vivo* relevant conditions. The last proposition to improve human pro-resolving macrophage understanding is to generate an *in vitro* model allowing to induce macrophage reprogramming, and to study their differentiation *in vitro*. This approach allows to decipher mouse pro-resolving macrophages and efferocytosis using hamster phagocytes and apoptotic human cells (57, 58). We have recently described and validated a complete human system, suitable for RNA sequencing studies, in which apoptotic neutrophils are co-cultured with human MDM (59). This model may allow the identification of pathways involved in pro-resolving macrophage reprogramming.

## Author Contributions

PS wrote the manuscript. PS and CC made the figure. CC, MM, and TC reviewed and edited the manuscript. All authors contributed to the article and approved the submitted version.

## Funding

This work is supported by the Agence Nationale de la Recherche (ANR) under the program “Investissements d’Avenir” with reference ANR-11-LABX-0021-LipSTIC, by the Region Bourgogne Franche-Comté (support to LipSTIC LabEX), the MiMedI project funded by BPI France (grant No. DOS0060162/00), and the European Union through the European Regional Development Fund of the Region Bourgogne-Franche-Comte (grant No. FC0013440).

## Conflict of Interest

The authors declare that the research was conducted in the absence of any commercial or financial relationships that could be construed as a potential conflict of interest.
